# The Covariance Adjustment Approaches for Combining Incomparable Cox Regressions Caused by Unbalanced Covariates Adjustment: A Multivariate Meta-Analysis Study

**DOI:** 10.1155/2015/801031

**Published:** 2015-09-01

**Authors:** Tania Dehesh, Najaf Zare, Seyyed Mohammad Taghi Ayatollahi

**Affiliations:** ^1^Department of Biostatistics, Faculty of Medicine, Shiraz University of Medical Sciences, P.O. Box 71345-1874, Shiraz, Iran; ^2^Department of Biostatistics, Infertility Research Center, Shiraz University of Medical Sciences, P.O. Box 71345-1874, Shiraz, Iran

## Abstract

*Background*. Univariate meta-analysis (UM) procedure, as a technique that provides a single overall result, has become increasingly popular. Neglecting the existence of other concomitant covariates in the models leads to loss of treatment efficiency. Our aim was proposing four new approximation approaches for the covariance matrix of the coefficients, which is not readily available for the multivariate generalized least square (MGLS) method as a multivariate meta-analysis approach.* Methods*. We evaluated the efficiency of four new approaches including zero correlation (ZC), common correlation (CC), estimated correlation (EC), and multivariate multilevel correlation (MMC) on the estimation bias, mean square error (MSE), and 95% probability coverage of the confidence interval (CI) in the synthesis of Cox proportional hazard models coefficients in a simulation study.* Result*. Comparing the results of the simulation study on the MSE, bias, and CI of the estimated coefficients indicated that MMC approach was the most accurate procedure compared to EC, CC, and ZC procedures. The precision ranking of the four approaches according to all above settings was MMC ≥ EC ≥ CC ≥ ZC.* Conclusion*. This study highlights advantages of MGLS meta-analysis on UM approach. The results suggested the use of MMC procedure to overcome the lack of information for having a complete covariance matrix of the coefficients.

## 1. Introduction

Meta-analysis is widely accepted as a systematic combination procedure using available evidence of independent studies for the purpose of a single overall result for the treatment of interest. This statistical procedure is becoming increasingly popular with medical researchers, particularly in clinical trials and survival analysis [1, 2]. Survival analysis is performed when the time is as the outcome variable and the Cox proportional hazard model is a famous procedure in this field [3]. Meta-analysis attempts to achieve a comprehensive result due to two procedures based on data availability: individual patient data (IPD) meta-analysis or aggregate patient data (APD) meta-analysis. It means that if individual's data from different studies are available, IPD is preferred, but if it is impossible to assemble the individual's raw data, then the APD should be chosen for combining the results of different studies. An IPD procedure requires individual data collection, whereas APD is based on summarized data that is extracted from published reports. While APD results are not as desirable as IPD, the majority of meta-analysis work relies on APD due to time availability and financial restriction [4–7].

Selection of homogeneous and related studies that address the same question is the first difficult task in each APD meta-analysis and has been debated by many authors [4, 8]. An additional problem is the fact that primary studies have diverse methodology and design protocols, such as differences in sample size and different covariate adjustment [9]. Of course, different covariate adjustment in different studies is a troublesome issue in any meta-analysis. This mis djustment of covariates may occur because of a decision by the analyst. Two suggested ways that have been used to date are (i) restricting the analysis to those models that have exactly the same set of covariates or (ii) using all available studies but omitting some important covariates. Both suggested ways lead to a waste of integrating information. As we know, omitting important concomitant covariates in nonlinear models leads to estimation bias and misleading interpretation, while the power is also decreased for no treatment effect detection even in primary studies [10–12]. In the Cox model, this covariates' omission leads to estimation bias toward zero for treatment effects [13, 14].

Nowadays, in APD, almost all researchers attempt to achieve the result for the desired variable by UM and neglect the existence of other covariates in the model. This could be problematic in meta-analysis. Several researchers have encountered this problem. With the exception of the limited number of studies that propose synthesis slopes in linear regression models [15], the others did not propose applicable solution methods, especially in nonlinear model fields. To combine incomparable Cox regression models, Yuan and Anderson proposed two approaches in 2010 that use the concomitant covariates in combining incomparable Cox models but in a UM manner and focused on a single interested covariate rather than estimating the influences of other concomitant covariates [16].

A multivariate meta-analysis approach can be one of the best solutions to overcome the problem of combining incomparable models by accounting for existences of all covariates in a meta-analysis simultaneously [17]. By using this approach in meta-analysis, no covariate or information is omitted, which leads to a more complete model with the estimated influences of all covariates to date. In addition to saving information on all covariates, which had previously used significant financial collection expenses in the primary studies, a complete model can be helpful in taking more confident therapeutic decisions. A useful and simple procedure in the multivariate meta-analysis is a generalized least square (GLS) estimation method. However, performing this method as a meta-analysis procedure has some difficulties due to lack of key information required [15, 17].

The main objective of this study is first to apply a MLGS method of synthesis of incomparable Cox regression slopes in order to have a complete Cox proportional hazard model with the effects of all available covariates and then to propose four new approaches to approximate the covariance matrix of coefficients as a necessary part of MGLS performance. We evaluated these four new proposed approaches by some statistical features. This paper also provides a general insight into the advantages of MGLS estimation over routine UM procedures that are usually used in meta-analysis.

## 2. Materials and Methods

### 2.1. Review of Conventional Univariate Meta-Analysis

The popular and used univariate meta-analysis approaches are based on weighted mean estimator described by A. Whitehead and J. Whitehead [18] and DerSimonian and Laird [1]. In a meta-analysis, we encounter two approaches, fixed effect and random effect analysis. If all the studies are assumed to have the same common treatment effects and differences between the studies are assumed to be due to chance, the fixed effect procedure is suitable, but the final result cannot be generalized to the population [19]. In the random effect modeling approach, the overall study variations are divided in to two parts: between-study variation represented by a random term (*a*_*i*_) and within-study variation represented by (*ε*_*i*_) in the model. The results of random effect meta-analysis can be generalized to the population. The random term (*a*_*i*_) is assumed to have a normal distribution with mean zero and unknown variance  *τ* ^2^. *ε*_*i*_ is called measurement error and has a normal distribution with zero mean and known variance *σ*_*i*_^2^. Hence, if we denote by *θ*_*i*_ the treatment effect or estimated log hazard ratio of the *i*th study (*i* = 1,…, *k*) and *θ* is the true overall effect, then a simple model is written as ${\hat{\theta }}_{i}=\theta +\varepsilon_{i}$ (fixed effects model) and ${\hat{\theta }}_{i}=\theta +a_{i}+\varepsilon_{i}$ (random effects model). We assume that ${\hat{\theta }}_{i}$ is approximately distributed as *N*(*θ*, *σ*_*i*_^2^).

In conventional weighted mean approaches, using the reciprocal of variance estimation as the weight, the total *θ* is estimated as $\hat{\theta }=\sum_{i=1}^{k}w_{i}{\hat{\theta }}_{i}/\sum_{i=1}^{k}w_{i}$ with $var(\hat{\theta })=(1/\sum_{i=1}^{k}w_{i})$, where *w*_*i*_ is the weight given to study *i*.

An approximate of *w*_*i*_ under the fixed effects assumption is *w*_*i*_ = 1/*σ*_*i*_^2^ and, under the random effects, reciprocal of between-study variances is added to the weights: $w_{i}={\left(w_{i}^{-1}+{\hat{\tau }}^{2}\right)}^{-1}$. Thus, studies with smaller variance are given more weight than those with large variances. Attribution of any weight to individual study is permitted, but since this weight is reported to provide the highest precision for total treatment effect [20], almost all meta-analysis researchers have used it.

The famous statistics to test the homogeneity of treatment effect across all studies is Cochran' *Q* statistic [21], which is constructed based on differences between the pooled estimate effect and each study effect.

Under the null hypothesis of homogeneity of treatment effect across all studies, *θ*_1_ = *θ*_2_ = ⋯*θ*_*k*_, *Q* follows *χ* ^2^ distribution with (*k* − 1) degrees of freedom. *Q* test is reported to have a low power, especially when the number of primary studies is small [22].

Another measurement of heterogeneity is provided by referring to *I* ^2^ index as follows: *I* ^2^ = Max(0, (*Q* − (*k* − 1))/*Q* × 100%). The percentage of total variability due to between-study variability is interpreted by *I* ^2^ index. Recently, *I* ^2^ has become more well known in meta-analysis, because it indicates the percentage magnitude of heterogeneity [23].

### 2.2. MGLS in Meta-Analysis with Cox Models

Recently, the synthesis of regression slopes has received more attention in meta-analysis [24]. We illustrate synthesis of the incomparable Cox regression model slopes based on MGLS approach, presented by Becker and Wu previously [15].

Suppose that we have *p* covariates in whole *k* studies that participate in meta-analysis and suppose that *X*_1_ is the interested variable that existed in *k* studies and the other *p* − 1 covariates exist only for adjustment. It means that each study has some of the covariates, not all of them. Furthermore, there is no treatment by covariate interaction. A full adjustment Cox model is as follows:

(1) $$\begin{array}{c} h\left(t,X\right)=h_{0}\left(t\right)\exp\left(\beta_{1}x_{1}+\cdots +\beta_{p}x_{p}\right). \end{array}$$

For convenience, we illustrated MGLS estimation method in combining incomparable Cox models. Suppose that we want to combine the results of three studies of Cox models in MGLS approach and we have four covariates besides *X*_1_ which is the interested variable in whole *k* studies. In the first study, we have the coefficients of these covariates (*X*_1_, *X*_2_, *X*_3_, *X*_4_), in the second study (*X*_1_, *X*_3_, *X*_5_), and in the last one (*X*_1_, *X*_5_). For each covariate, an indicator variable was defined that takes the value of 1 if those covariates exist in the study and zero otherwise. Here we assume coefficients as responses and construct a multivariate format. In fact, we want to estimate the influence of all covariates, not the hazards in different times, so the multivariate approach is acceptable. The MGLS for the above example can be shown in a matrix form as follows:

(2) $$\begin{array}{c} \left[\begin{array}{c} b_{11}\\ b_{21}\\ b_{31}\\ b_{41}\\ \cdots \\ b_{12}\\ b_{32}\\ b_{52}\\ \cdots \\ b_{13}\\ b_{53} \end{array}\right]=\left[\begin{array}{ccccc} 1 & 0 & 0 & 0 & 0\\ 0 & 1 & 0 & 0 & 0\\ 0 & 0 & 0 & 1 & 0\\ 0 & 0 & 0 & 1 & 0\\ \cdots & \cdots & \cdots & \cdots & \cdots \\ 1 & 0 & 0 & 0 & 0\\ 0 & 0 & 1 & 0 & 0\\ 0 & 0 & 0 & 0 & 1\\ \cdots & \cdots & \cdots & \cdots & \cdots \\ 1 & 0 & 0 & 0 & 0\\ 0 & 0 & 0 & 0 & 1 \end{array}\right]\left[\begin{array}{c} \beta_{1}\\ \beta_{2}\\ \beta_{3}\\ \beta_{4}\\ \beta_{5} \end{array}\right]+\left[\begin{array}{c} \varepsilon_{11}\\ \varepsilon_{21}\\ \varepsilon_{31}\\ \varepsilon_{41}\\ \cdots \\ \varepsilon_{12}\\ \varepsilon_{32}\\ \varepsilon_{52}\\ \cdots \\ \varepsilon_{13}\\ \varepsilon_{53} \end{array}\right]+\left[\begin{array}{c} a_{11}\\ a_{21}\\ a_{31}\\ a_{41}\\ \cdots \\ a_{12}\\ a_{32}\\ a_{52}\\ \cdots \\ a_{13}\\ a_{53} \end{array}\right]. \end{array}$$

In above matrix form, *b*_*ij*_ is the coefficient of covariate *j* in study *i* (*i* = 1,…, *k*), (*j* = 1,…, *p*). An alternative writing of the above model is *b* = *Hβ* + *e* + *a*, where *b* is a vector of all covariate coefficients from entire studies, *H* is a matrix that contains 1 and 0 in each row representing covariate existence in each study and the columns contain all covariates (here *p* covariates) in meta-analysis, *e* is a vector of sampling errors, and *a* is a vector of random effects that is computed from between-coefficient variability. As we know, the best linear unbiased estimator of *β* with MGLS procedure is $\hat{\beta }={\left(H\Sigma^{-1}H\right)}^{-1}H\Sigma^{-1}b$ and the covariance matrix of $cov\left(\hat{\beta }\right)=\;({H\Sigma^{-1}H)}^{-1}$ with a large sample $\hat{\beta }$ is asymptotically normally distributed: $\hat{\beta }\;\sim N$ ($\beta ,cov(\hat{\beta })$). If we consider the *q*th diagonal element of $cov(\hat{\beta })$ as *c*_*qq*_ and if *b* is a multivariate normal, then the confidence interval and hypothesis test for each *β* is available: ${\hat{\beta }}_{i}\pm z_{1-\alpha /2}\sqrt{c_{qq}}$, where *z*_1−*α*/2_ is the upper tail 1 − *α*/2 critical value of standard normal distribution.

A homogeneity test of all coefficients across studies under the normality assumption for *b* is given as follows.

$Q=(b-H\hat{\beta })\Sigma^{-1}(b-H\hat{\beta })$ which has a large sample *χ* ^2^ with (*k* − 1)*p* degrees of freedom, where *k* and *p* are the number of studies and covariates in all studies, respectively.

As we can observe clearly, all the estimations depend on the blockwise diagonal covariance matrix of coefficients (Σ). Without having a complete coefficients covariance matrix (Σ) or a suitable estimated coefficients covariance matrix (*S*), all MGLS estimates have computation problems. For instance, the covariance matrix of coefficients in the above example is as follows:

(3) $$\begin{array}{c} \Sigma =\left[\begin{array}{ccc} \left[\begin{array}{cccc} \delta_{1}^{2} & \delta_{12} & \delta_{13} & \delta_{14}\\ \delta_{21} & \delta_{2}^{2} & \delta_{23} & \delta_{24}\\ \delta_{31} & \delta_{32} & \delta_{3}^{2} & \delta_{34}\\ \delta_{41} & \delta_{42} & \delta_{43} & \delta_{4}^{2} \end{array}\right] & \begin{array}{c} 0\\ 0\\ 0\\ 0 \end{array} & \begin{array}{c} 0\\ 0\\ 0\\ 0 \end{array}\\ \begin{array}{c} 0\\ 0\\ 0 \end{array} & \left[\begin{array}{ccc} \delta_{1}^{2} & \delta_{13} & \delta_{15}\\ \delta_{31} & \delta_{3}^{2} & \delta_{35}\\ \delta_{51} & \delta_{53} & \delta_{5}^{2} \end{array}\right] & \begin{array}{c} 0\\ 0\\ 0 \end{array}\\ \begin{array}{c} 0\\ 0 \end{array} & \begin{array}{c} 0\\ 0 \end{array} & \left[\begin{array}{cc} \delta_{1}^{2} & \delta_{15}\\ \delta_{51} & \delta_{5}^{2} \end{array}\right] \end{array}\right]=\left[\begin{array}{ccc} \mathrm{cov}\left(b_{1}\right) & 0 & 0\\ 0 & \mathrm{cov}\left(b_{2}\right) & 0\\ 0 & 0 & \mathrm{cov}\left(b_{3}\right) \end{array}\right]. \end{array}$$

*b*_1_, *b*_2_, and *b*_3_ are three covariate coefficient vectors of three primary studies. The major limitation and problem that has been presented previously is lack of actual complete coefficients covariance matrixes from primary studies [17, 19]. The multivariate coefficients covariance matrix is a blockwise diagonal that includes the variance of covariate coefficients on its diagonal, which can almost always be found in the Cox model results and between-coefficients covariances on off-diagonal parts which are rarely reported even in recently published papers.

We attempted to propose approximations for the covariance of covariate coefficients and construct a covariance matrix as close as possible to the actual Σ to have the MGLS coefficients estimates finally.

### 2.3. Four New Approaches

Suppose that, in one of the primary Cox models, we have coefficients *b*_1_ and *b*_3_. From basic statistical laws, the covariance of these two coefficients can be obtained by  cov(*b*_1_, *b*_3_) = Corr(*b*_1_, *b*_3_)SE(*b*_1_)SE(*b*_3_); this can be generalized to all coefficients in whole studies:

(4) $$\begin{array}{c} \mathrm{cov}\left(b_{ij},b_{ij}\right)=\text{Corr}\left(b_{ij},b_{ij}\right)\text{SE}\left(b_{ij}\right)\text{SE}\left(b_{ij}\right) \end{array}$$

(*i* = 1,…, *k*), (*j* = 1,…, *p*). So if we have a correlation value between each paired coefficients, the covariance can be calculated simply. Therefore, we propose four approaches for the correlation calculation to approximate coefficients covariances, which is one of the main purposes of this study.

#### 2.3.1. Zero Correlation (ZC)

We can assume that the authors during primary studies reported a very qualified model in the initial studies that completely checked for lack of multicollinearity. We can ignore the correlations and take them as zero in (4), so *S* matrixes are a diagonal that has only actual available variance of the coefficient on its diagonal.

#### 2.3.2. Common Correlation (CC)

Lack of multicollinearity is rather unlikely, even when considered optimistic. In a very qualified model, a little multicollinearity is unavoidable. Therefore, we can take a little common correlation value among all coefficients in all *k* studies. For example, we can assume  Corr(*b*_*ij*_, *b*_*ij*_) = 0.3. This assumption does not have any influence in calculation of *S*, because it is a common value for all coefficients. By substitution of this common value in (4), all covariances can be calculated.

#### 2.3.3. Estimated Correlation (EC)

In this approach, we can extract all similar coefficients from all studies that participated in meta-analysis. After extracting, we must put similar coefficients in the same *b*_*j*_ vectors (*j* = 1, …, *p*). Therefore, we have some *b*_*j*_ vectors, but with different lengths, because some of the covariates may participate in fewer studies than others. Then, the correlation between these vectors can be used as the correlations part in (4). The benefit of this approach is the fact that we use completely real available information in correlation computation instead of zero or common values based on educated guesses that have been used in two previous approaches. This approach also has a drawback and limitation; it is useful only in those meta-analyses that have many primary studies. The reason is described in the following paragraph.

One important point that should be paid attention to is that we must extract the covariate coefficients that has similar concomitant coefficients along themselves in the same study. For example, if there are two studies with (*b*_1_, *b*_2_, *b*_3_) and (*b*_1_, *b*_3_, *b*_5_), these two *b*_1_ coefficients cannot be in the same *b*_1_ vector because they have different concomitant covariate coefficients with each other that have influence on their values. For a more detailed illustration, assume that we have only eight primary studies in a meta-analysis where *b*_1_ exists in all of them as a desired variable and the other four covariates (2, 3, 4, and 5) participated randomly in each of the models as follows:

 
*Study 1*: (*b*_1_, *b*_5_, *b*_4_).
 
*Study 2*: (*b*_1_, *b*_2_, *b*_5_)*∗*.
 
*Study 3*: (*b*_1_, *b*_3_, *b*_4_).
 
*Study 4*: (*b*_1_, *b*_5_, *b*_3_).
 
*Study 5*: (*b*_1_, *b*_2_, *b*_5_).
 
*Study 6*: (*b*_1_, *b*_2_, *b*_5_)*∗*.
 
*Study 7*: (*b*_1_, *b*_4_, *b*_3_).
 
*Study 8*: (*b*_1_, *b*_2_, *b*_5_)*∗*.

In the above example, we have only 3 *b*_*i*_ vectors, *b*_1_ = (*b*_12_, *b*_15_, *b*_18_), *b*_2_ = (*b*_22_, *b*_25_, *b*_28_), and *b*_5_ = (*b*_52_, *b*_55_, *b*_58_), the elements of which come from the second, fifth and eighth studies, so the three correlations coefficients are computable. As we can observe, the other coefficients in other studies have different concomitant coefficients with themselves; therefore, they cannot be in the same vectors and cannot have correlation. In fact, in this example, we have five coefficients in whole 8 studies (*b*_1_, *b*_2_, *b*_3_, *b*_4_, *b*_5_), but we can calculate the correlations, only between three of them (*b*_1_, *b*_2_, *b*_5_). For the other coefficients, we take their correlation values as zero. Logically, when we have more studies, this problem does not occur and we can obtain *b*_*i*_ vectors with longer lengths for all coefficients and therefore all correlations are calculable.

#### 2.3.4. Multivariate Multilevel Correlation (MMC)

The final suggestion is to look at the studies and covariate coefficients as a multivariate multilevel model. Goldestein has explained that multivariate response data are conveniently incorporated into multilevel models by creating an extra level below the original level 1 to define multivariate structure. There are several interesting features of this model. This model does not have level 1 variability because level 1 exists only to define multivariate structure. Level 2 variances and covariance are the between-studies variation. Another important feature is the fact that the multivariate multilevel estimates are statistically efficient even where some responses are missing in meta-analysis of some studies that do not have some of the coefficients [25]. We have two levels: covariates coefficients as level 1 are nested in studies as level 2. Each response was formulated as follows:

(5) $$\begin{array}{c} b_{ij}=b_{j}+u_{ij}, \end{array}$$

where *i* is the index for the study (*i* = 1,…, *k*) and *j* is for covariates in all studies (*j* = 1,…, *p*) and *u*_*ij*_ is the random term of the responses. We have a covariance and a correlation matrix for the random part between all *p* covariates. Each response or each coefficient was formulated separately. For example, for two coefficients, the formulas are as follows:

(6) $$\begin{array}{c} b_{i1}=b_{1}+u_{i1}\\ b_{i2}=b_{2}+u_{i2}. \end{array}$$

So these random parts are as follows:

(7) $$\begin{array}{c} \left[\begin{array}{c} u_{i1}\\ u_{i2} \end{array}\right]\sim N\left(0,\varphi \right),\;\varphi =\left[\begin{array}{cc} \delta_{i1}^{2} & \delta_{12}\\ \delta_{21} & \delta_{i2}^{2} \end{array}\right]. \end{array}$$

From this covariance matrix, the correlations between coefficients can be calculated and substituted in (4) and then *S* matrix obtained finally.

MLwiN 2.3 is software for doing multivariate multilevel analysis that is linked to R software recently and all above calculations can be done using this software.

### 2.4. Mean Absolute Percentage Error (MAPE)

Several statistics for model checking are available, but when we have lack of sufficient information, for example, in a meta-analysis, MAPE can be a suitable choice.

The coverage probability of 95% of Wald (W) and Bonferroni (B) CI was also calculated as another evidence for comparing the efficiencies of the four new approaches. The number of time that all 95% BCI cover real coefficients values of all coefficients simultaneously among 2000 simulations were also calculated for each of the four approaches. The MAPE and WCI and BCI formulas are presented in the Appendix.

### 2.5. Simulation Studies

We explore some statistical properties of four new approaches in terms of MSE, estimation bias, the amount of reduction in MAPE, and the coverage probability of 95% WCI and BCI in R software. We simulated survival times as the first required part in a simulation of the Cox model based on the procedure described by Blender and his coworkers in 2011 and Austin in 2010 [26, 27]. We generated a Cox model with five covariates similar to that observed in the male breast cancer clinical trials, as an example of a rare cancer for which we had survival data on. Our simulation design for obtaining Cox beta coefficients followed the procedure used by Yuan and Anderson in 2010 [16]. We assumed and generated Cox models with five covariates.

(8) $$\begin{array}{c} h\left(x,t\right)=h_{0}\left(t\right)\exp\left(\beta_{1}X_{1}+\beta_{2}X_{2}+\beta_{3}X_{3}+\beta_{4}X_{4}+\beta_{5}X_{5}\right). \end{array}$$

To simulate covariates similar to those observed in a male breast cancer, *X*_1_ was generated from a Bernoulli distribution with *P* = 0.5 to represent a treatment indicator, *X*_2_ was generated as a covariate of centered age from a normal distribution with mean 0 and variance 100, *X*_3_ was generated as a covariate of tumor size of *χ* ^2^ distribution with four degrees of freedom, and *X*_4_ was generated from a Bernoulli variable with *P* = 0.5 as an indicator of auxiliary lymph node involvement. *X*_5_ stands for the number of lymph nodes involved with male breast cancer, generated from an exponential distribution with the rate of 1/4, rounded up to an integer and a modification applied to them because a negative nodal status (*X*_5_) would occur in roughly 40% of patients. To generate the survival times, the values coefficients of *X*_1_ to *X*_5_ were set to

(9) $$\begin{array}{c} \beta =\left(\beta_{1},\beta_{2},\beta_{3},\beta_{4},\beta_{5}\right)=\left(-0.2,-0.1,0.1,0.54,0.7\right) \end{array}$$

based on real values similar to those observed in male breast cancer patients. *X*_1_ exists in all *k* studies, but other covariates were chosen without replacement from *X*_2_ to *X*_5_. The survival times were randomly censored with probability 0.1. The baseline failure time is generated from an exponential distribution with *λ*_0_ = 0.2. The number of primary studies was set in turn to 20, 25, 30, 35, 40, and 45. The number of patients in each study was randomly picked up from 100 to 500 and survival times for each study were censored with probability randomly chosen between 0.1 and 0.4.

After extracting covariates coefficients from different simulated Cox studies, *H* matrix was constructed by arranging all *H*_*i*_ from different studies under each other.

*S* matrix was constructed based on the four proposed methods and substituted in the MGLS estimation formula. If the heterogeneity of studies was rejected, then the variance between each pair of coefficients (as a random parts of the model) is added to the diagonal elements of *S* matrix. Then the final covariate coefficient was estimated from the MGLS procedure. We generated 2000 random data sets for each simulation scenario and all statistical settings are the average of these 2000 simulations. The multivariate multilevel covariance matrixs were calculated by the R2MLwiN package in R software, like all other simulation procedures.

## 3. Results

Table 1 shows the bias, standard deviation (SD), and MSE of four proposed methods under different number of studies (*k* from 20 to 45), each for 2000 simulations. The result of ZC method is similar to the traditional weighted mean meta-analysis that is used routinely in meta-analysis work, especially for *β*_1_ that exists in all studies.

The first notable point that can be seen in this table is the fact that multivariate methods (CC, EC, and MMC) are preferable to the conventional weighted mean method (ZC) for *β*_1_, according to MSE, SD, and bias. In terms of the lowest MSE, bias, and also SD for the all estimated coefficients, the four methods are generally ranked as MMC ≤ EC ≤ CC ≤ ZC.

**Figure 1:**
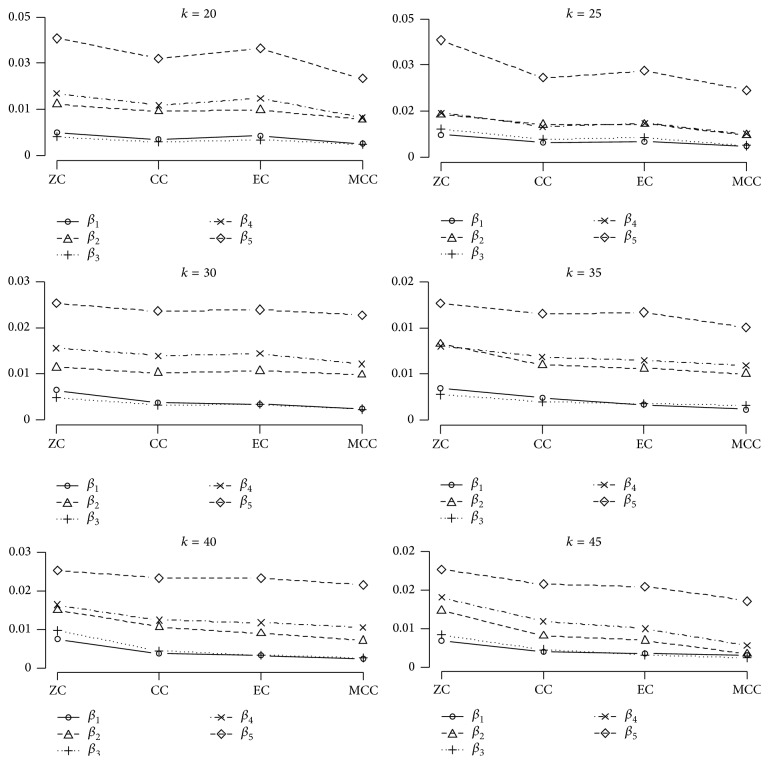
Comparisons of the estimated MSE in the four proposed methods with different numbers of studies. The horizontal axis indicates the four proposed methods. ZC: zero correlation; CC: common correlation; EC: estimated correlation; MMC: multivariate multilevel correlation. Each point on the graph is averaged over 2000 simulation realizations.

**Figure 2:**
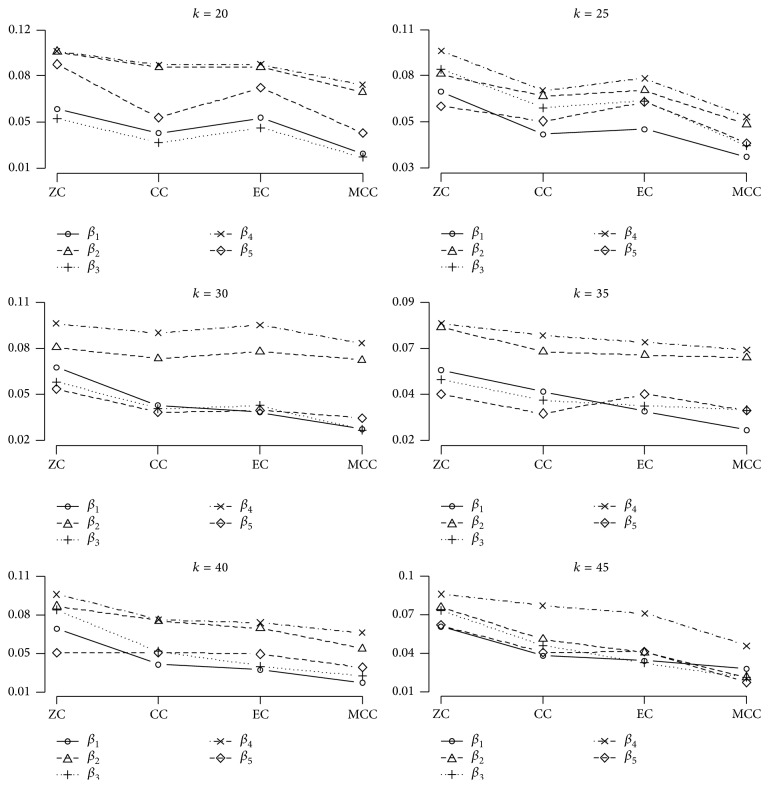
Comparisons of the estimated bias in the four proposed methods with different numbers of studies. The horizontal axis indicates the four proposed methods. ZC: zero correlation; CC: common correlation; EC: estimated correlation; MMC: multivariate multilevel correlation. Each point on the graph is averaged over 2000 simulation realizations.

Figures 1 and 2 illustrate the above results again more clearly.

As we can observe in Figure 1, the MMC method has a much smaller MSE relative to the other three methods for all MGLS estimated coefficients.

Figure 2 shows that the MMC method has almost the smallest estimated bias values among the four proposed methods for all MGLS estimated coefficients, too. When the number of studies is rather small (relative to our number of studies) (*k* = 20, 25), the lowest value of both mentioned statistics belongs to the MMC and CC, respectively; as *k* increased (*k* = 30, 35), the bias and MSE of the EC method decreased moderately and its values became as close as CC method. Indeed, when *k* was larger (*k* = 40, 45), EC method showed smaller MSE and bias than CC and its results became closer to those of the MMC method. This can be seen obviously in Figure 1 for the MSE curves.

Table 2 shows the coverage probability for 95% WCI and 95% BCI for all coefficients and the percentage of times that the simultaneous BCIs cover all five true values of beta coefficients in the whole of 2000 simulations (%BCI).

Our findings reveal that all the 95% confidence intervals that were constructed with the MMC proposed method had a higher probability of covering true values of beta coefficient, both in WCI and BCI, which are highlighted by bold font in Table 2.

Indeed, when the numbers of studies were small, CC showed better coverage, but as *k* increased, EC could substitute CC again. In addition, according to the MAPE statistic for the adequacy of model checking, the MMC method showed the lowest value among the four methods in different number of studies and therefore had a better model fitting.

## 4. Discussion

In summary, our results showed that, based on the estimation bias, MSE, coverage probability for 95% CI, and MAPE value, the MMC method is more efficient than the other three methods, followed by CC for small *k* and EC for large *k*. In the APD setting, where we only observe summarized model information from different studies, UM as a completely well-known meta-analysis procedure has a reputation and universal position in statistical literature. Weighted mean has the highest usage among other meta-analysis procedures. In this method, the weights are factors that bring primary study characteristics into meta-analysis results. In fact, they are representative of initial study features. In this established popular procedure, the influences of concomitant covariates that exist only for adjustment and do not have main role are often neglected. In a meta-analysis, combining estimates of different studies that are adjusted for different sets of covariates is problematic. Therefore, besides the loss of their information, neglecting of these covariates introduces bias and inefficiency in interpretation of results, especially in nonlinear models [9–13].

To the best of our knowledge, no previous study has completely paid attention to a multivariate procedure in meta-analysis in order to solve the above problem [15, 16]. Yuan takes into account the existence of other concomitant variables in incomparable Cox models but did not estimate the comprehensive effect for them. He proposed two approaches for adjusting the influences of all covariates in estimating the effect of the variable of interest. Despite his attention to a single variable, the two proposed methods showed more precise effects than the familiar UM method. Becker and Wu also used the GLS multivariate method as a meta-analysis approach but did not express an applicable way for constructing the coefficient covariance matrix as a necessary part in applying GLS for meta-analysis [15].

Our findings also show the advantages of using a multivariate approach on UM. The MGLS has this benefit over Yuan method in that it estimates all coefficients affect simultaneously not only the variable coefficient of interest. As we can observe from Tables 1 and 2 and Figures 1 and 2, ZC, which gives the same result as UM, especially for *b*_1_ coefficient that exists in all *k* studies, has the highest bias, MSE, MAPE, and the minimum 95% CI coverage among other mentioned multivariate procedures. Therefore, the MGLS procedure is preferred to UM in this situation due to its greater precision even for  *b*_1_.

Our simulation results in all tables and figures show that, according to the computed statistics here, MMC gives more accurate final covariate coefficient estimations for all five covariates. The ZC procedure is not recommended because ignoring the correlations between coefficients is not logical at all. Of course, this procedure is the simplest one as recommended previously by Becker and Wu [15], but as we can see it leads to the lowest precision in the final results. Little multicollinearity even in the precise model fitting cannot be completely neglected.

The CC procedure, which used a common correlation value between all coefficients, shows more precise results than the ZC approach. Of course, a common correlation value does not have any special influence on the final results, because it is common and fixed value between all coefficients, but its usage leads to applying real coefficient variances in estimations. It is completely acceptable and logical that the real variance application can improve the precision of the result compared to educated guesses, like zero in ZC.

The CC procedure has better results than EC when the numbers of studies are small. This reduced precision in EC is due to a large number of studies which its estimation needs. As we mentioned in Materials and Methods, the EC procedure needs similar studies according to covariates to find the same coefficient with the same concomitant covariates along them. In fact, coefficients cannot be considered as variables because they are effects. Their omission influences the other coefficient calculations in primary studies. As the results indicated, when the number of studies increased, EC precedes CC in precision. This result is also acceptable, because the EC computation relies more on real information that is available from initial studies compared to the CC procedure. As we move toward real available information in our procedures, estimations become more precise. This point shows the importance of actual information that when we use more realistic data, we observe more accurate results.

Our study results emphasized the fact that MMC is the most precise and accurate covariance approximation method in MGLS meta-analysis among the four new proposed methods. The MMC procedure uses a multivariate multilevel technique in coefficient correlation estimation. The advantages of this procedure are the fact that real available information is completely used in a logical manner for correlation estimation. In fact, the MMC procedure looks at initial coefficients as responses that are nested in studies as the second level. This is the logical way of consideration and the correlations are completely estimated by a familiar logical procedure (multivariate multilevel method). In this study, MMC is suggested as the recommended procedure to overcome the lack of a covariance matrix.

As the MMC procedure needs specialized packages which may not be readily available, we suggest CC for a small number of *k* (*k* ≤ 30) and EC for a large number (*k* ≥ 35) as a suitable substitution.

Of course, the quality of all meta-analysis procedures can improve by encouraging authors to report more information, like coefficient covariance matrix. In that case, there will be no need for these approximate procedures in future. In fact, undoubtedly due to the huge number of published papers, meta-analysis will become a more useful method in future and authors should believe it and be more forthcoming in reporting of information.

## 5. Conclusion

Combining Cox regression coefficients in a multivariate meta-analysis manner, besides offering to represent an overall treatment effect, gives us a full Cox model as a complete risk factor model in medical decision making. The MMC is the accurate procedure in the covariance approximation in applying MGLS in meta-analysis.

## Figures and Tables

**Table 1:** Bias, SD, and MSE of each beta coefficient in different model for different number of studies (*k*).

*k*	Coef.	ZC			CC			EC			MMC		
		Bias	SD	MSE	Bias	SD	MSE	Bias	SD	MSE	Bias	SD	MSE
20	β_1_	0.056	0.046	0.005	0.038	0.034	0.003	0.050	0.039	0.004	**0.023**	**0.021**	**0.001**
	β_2_	0.099	0.077	0.016	0.088	0.074	0.013	0.088	0.077	0.014	**0.070**	**0.072**	**0.010**
	β_3_	0.050	0.033	0.004	0.031	0.026	0.002	0.042	0.026	0.002	**0.020**	**0.016**	**0.001**
	β_4_	0.099	0.099	0.019	0.089	0.086	0.015	0.089	0.099	0.018	**0.045**	**0.072**	**0.011**
	β_5_	0.090	0.179	0.041	0.050	0.174	0.033	0.072	0.178	0.037	**0.040**	**0.155**	**0.030**

25	β_1_	0.071	0.029	0.006	0.046	0.027	0.003	0.049	0.028	0.003	**0.032**	**0.019**	**0.001**
	β_2_	0.082	0.083	0.014	0.069	0.072	0.009	0.072	0.069	0.010	**0.052**	**0.057**	**0.006**
	β_3_	0.085	0.029	0.008	0.062	0.019	0.004	0.066	0.023	0.005	**0.039**	**0.011**	**0.002**
	β_4_	0.096	0.072	0.015	0.072	0.063	0.009	0.080	0.064	0.010	**0.056**	**0.056**	**0.006**
	β_5_	0.063	0.198	0.043	0.054	0.159	0.028	0.066	0.164	0.031	**0.041**	**0.147**	**0.023**

30	β_1_	0.064	0.030	0.005	0.038	0.025	0.002	0.034	0.026	0.002	**0.022**	**0.018**	**0.001**
	β_2_	0.076	0.064	0.009	0.069	0.063	0.009	0.074	0.061	0.009	**0.068**	**0.060**	**0.008**
	β_3_	0.054	0.019	0.003	0.036	0.018	0.002	0.038	0.017	0.002	**0.022**	**0.015**	**0.001**
	β_4_	0.092	0.076	0.014	0.086	0.072	0.013	0.092	0.068	0.013	**0.079**	**0.066**	**0.011**
	β_5_	0.049	0.148	0.024	0.034	0.146	0.022	0.035	0.147	0.023	**0.030**	**0.144**	**0.022**

35	β_1_	0.055	0.026	0.04	0.043	0.021	0.002	0.032	0.017	0.001	**0.022**	**0.014**	**0.001**
	β_2_	0.079	0.062	0.010	0.065	0.051	0.007	0.064	0.050	0.007	**0.062**	**0.044**	**0.006**
	β_3_	0.050	0.018	0.003	0.039	0.016	0.002	0.035	0.014	0.001	**0.033**	**0.012**	**0.001**
	β_4_	0.081	0.057	0.009	0.075	0.052	0.008	0.071	0.053	0.008	**0.066**	**0.051**	**0.007**
	β_5_	0.042	0.119	0.016	0.031	0.116	0.014	0.042	0.113	0.014	**0.033**	**0.106**	**0.012**

40	β_1_	0.069	0.026	0.005	0.038	0.022	0.002	0.034	0.021	0.002	**0.023**	**0.016**	**0.001**
	β_2_	0.089	0.069	0.013	0.076	0.052	0.008	0.069	0.045	0.007	**0.052**	**0.049**	**0.005**
	β_3_	0.085	0.017	0.008	0.049	0.016	0.003	0.037	0.015	0.002	**0.029**	**0.013**	**0.001**
	β_4_	0.098	0.064	0.014	0.077	0.065	0.010	0.074	0.064	0.009	**0.066**	**0.064**	**0.008**
	β_5_	0.049	0.141	0.022	0.049	0.134	0.020	0.047	0.134	0.020	**0.036**	**0.132**	**0.019**

45	β_1_	0.061	0.026	0.004	0.039	0.023	0.002	0.035	0.021	0.002	**0.029**	**0.021**	**0.001**
	β_2_	0.076	0.073	0.011	0.051	0.054	0.006	0.041	0.053	0.004	**0.022**	**0.033**	**0.002**
	β_3_	0.073	0.019	0.006	0.047	0.018	0.002	0.033	0.016	0.001	**0.022**	**0.013**	**0.001**
	β_4_	0.086	0.082	0.014	0.077	0.052	0.009	0.071	0.045	0.007	**0.047**	**0.036**	**0.003**
	β_5_	0.062	0.128	0.020	0.041	0.123	0.017	0.042	0.121	0.016	**0.018**	**0.113**	**0.013**

The bias, SD, and MSE for MMC procedure in different coefficients are highlighted by bold font.

**Table 2:** MAPE, the coverage probability for 95% W and B, CI, and MSE and %BCI of each estimated beta coefficient in the four proposed methods for different number of studies (*k*).

*k*	Model	MAPE	%BCI	CI	β_1_	β_2_	β_3_	β_4_	β_5_
20	ZC	0.236	0.49	W	0.88	0.91	0.72	0.90	0.89
				B	0.74	0.84	0.59	0.83	0.99
	CC	0.229	0.63	W	0.91	0.95	0.87	0.94	0.99
				B	0.77	0.85	0.68	0.85	0.99
	EC	0.233	0.60	W	0.89	0.94	0.82	0.93	0.99
				B	0.72	0.84	0.60	0.82	0.98
	MMC	**0.220**	**0.65**	W	**0.92**	**0.95**	**0.91**	**0.94**	**0.99**
				B	**0.78**	**0.88**	**0.88**	**0.87**	**0.99**

25	ZC	0.218	0.51	W	0.86	0.90	0.75	0.90	0.98
				B	0.69	0.82	0.51	0.79	0.97
	CC	0.216	0.56	W	0.88	0.92	0.81	0.93	0.99
				B	0.74	0.84	0.60	0.82	0.98
	EC	0.217	0.52	W	0.86	0.91	0.73	0.89	0.98
				B	0.68	0.82	0.50	0.79	0.98
	MMC	**0.212**	**0.62**	W	**0.89**	**0.92**	**0.78**	**0.94**	**0.99**
				B	**0.75**	**0.85**	**0.65**	**0.84**	**0.99**

30	ZC	0.219	0.52	W	0.81	0.90	0.64	0.85	0.96
				B	0.63	0.82	0.39	0.78	0.97
	CC	0.214	0.63	W	0.86	0.94	0.71	0.90	0.98
				B	0.68	0.83	0.49	0.81	0.98
	EC	0.215	0.62	W	0.84	0.93	0.68	0.89	0.98
				B	0.66	0.82	0.47	0.79	0.98
	MMC	**0.206**	**0.68**	W	**0.88**	**0.95**	**0.75**	**0.91**	**0.99**
				B	**0.68**	**0.84**	**0.51**	**0.82**	**0.98**

35	ZC	0.208	0.57	W	0.79	0.90	0.61	0.90	0.96
				B	0.56	0.78	0.36	0.78	0.97
	CC	0.198	0.66	W	0.83	0.92	0.70	0.93	0.98
				B	0.64	0.82	0.44	0.82	0.98
	EC	0.197	0.68	W	0.84	0.92	0.72	0.94	0.98
				B	0.65	0.84	0.46	0.87	0.98
	MMC	**0.194**	**0.71**	W	**0.85**	**0.94**	**0.81**	**0.94**	**0.99**
				B	**0.67**	**0.84**	**0.54**	**0.83**	**0.98**

40	ZC	0.197	0.52	W	0.76	0.90	0.56	0.89	0.96
				B	0.57	0.81	0.39	0.77	0.95
	CC	0.174	0.61	W	0.87	0.95	0.76	0.95	0.98
				B	0.75	0.87	0.53	0.85	0.98
	EC	0.170	0.67	W	0.89	0.98	0.78	0.95	0.99
				B	0.79	0.88	0.58	0.87	0.98
	MMC	**0.162**	**0.69**	W	**0.92**	**0.98**	**0.85**	**0.96**	**0.99**
				B	**0.85**	**0.88**	**0.61**	**0.89**	**0.99**

45	ZC	0.201	0.27	W	0.73	0.89	0.47	0.89	0.92
				B	0.52	0.77	0.22	0.75	0.97
	CC	0.177	0.55	W	0.86	0.92	0.73	0.94	0.98
				B	0.72	0.83	0.46	0.84	0.97
	EC	0.168	0.68	W	0.88	0.94	0.78	0.95	0.98
				B	0.73	0.85	0.48	0.84	0.98
	MMC	**0.162**	**0.73**	W	**0.90**	**0.95**	**0.82**	**0.95**	**0.99**
				B	**0.89**	**0.86**	**0.54**	**0.90**	**0.99**

The MAPE and the true coverage probability for 95% W and B and %BCI values for MMC procedure are highlighted by bold font.

## Appendix

The mean absolute percentage error (MAPE) function is as follows:

(A.1) $$\begin{array}{c} \text{MAPE}=\frac{1}{n}\overset{n}{\underset{t=1}{\sum }}\left|\frac{A_{t}-F_{t}}{A_{t}}\right|. \end{array}$$

*A*_*t*_ are observed values and *F*_*t*_ are forecasted values of each model. In our study, we take real coefficient values as observed value and GLS estimated coefficients as forecasted values of the model.

Wald confidence interval formula is as follows:

(A.2) $$\begin{array}{c} b_{l}\pm t\left(1-\frac{\alpha }{2},n-p\right). \end{array}$$

*b*_*l*_ is coefficient for each of *l* covariates and *n* and *p* are number of studies and covariates, respectively.  *α* is a critical value that is taken as 0.05 for 95% CI.

Simultaneous Bonferroni CI formula is as follows:

(A.3) $$\begin{array}{c} b_{l}\pm t\left(1-\frac{\alpha }{2g},n-p\right)S\left(b_{l}\right). \end{array}$$

*g* is the number of beta or covariates coefficients that we want to have 95% CI simultaneously and in our study it takes 5, becuase we have 5 covariates. *S*(*b*_*l*_) is the standard deviation of *b*_*l*_.  *n*, *p*, and *α* have the same expressions as the above WCI.

## Abbreviations

UM: Univariate meta-analysis
GLS: Generalized least square
ZC: Zero correlation
CC: Common correlation
EC: Estimated correlation
MMC: Multivariate multilevel correlation
CI: Confidence interval
MSE: Mean square error
MGLS: Multivariate generalized least square
APD: Aggregate patient data
IPD: Individual patient data
MAPE: Mean absolute percentage error.
